# XMR is a useful modality to guide, map and quantify the perfusion territories of coronary arteries

**DOI:** 10.1186/1532-429X-11-S1-O87

**Published:** 2009-01-28

**Authors:** Marcus Carlsson, Maythem Saeed

**Affiliations:** 1 Clinical Physiology, Lund, Sweden; 2 Dep of Radiology and Biomedical Imaging, San Francisco, CA USA

**Keywords:** Contrast Enhance, Coronary Catheterization, Perfusion Territory, Endovascular Catheter, Inversion Recovery Gradient

## Introduction

The effects of locally delivered angiogenic factors or stem cells are not well defined. Obstacles to effective angiogenic treatment have been the difficulties in providing clear delineation of the status and extent of the injury and the coronary artery perfusion territory.

## Purpose

This study aimed to determine the ability of selective injection of Gadolinium based MR contrast media (MR-CM) to map and quantify the territories of the major coronary arteries using first-pass perfusion (FPP) and early contrast enhanced (CE) MRI.

## Methods

**Figure 1:**
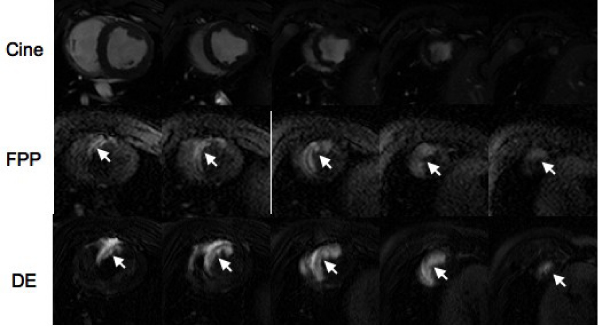
**Multislice MR images showing the LV on cine (top) and the LAD perfusion territory on corresponding FPP (middle) and early CE (bottom) MRI as hyperenhanced regions**.

Selective coronary catheterization (n = 16 pigs) was performed under X-ray and MRI fluoroscopy in an XMR-suite. Catheters were placed in LAD, circumflex or right coronary artery. The coronary perfusion territories were mapped by intracoronary injection of 6–10 ml 10% diluted MR-CM using a saturation-recovery gradient echo sequence for FPP images (TR/TE/flip = 4.5 ms/2.2 ms/20°, slice thickness = 10 mm) and an inversion recovery gradient echo sequence for early CE MRI (TR/TE/flip = 5 ms/2 ms/15°, shot interval = 2RR-intervals, slice thickness = 3–4 mm). Cine MRI was used to quantify LV mass. In 12 animals, the LAD was occluded by embospheres to create infarction. Infarct size was measured on delayed enhanced (DE) MRI after intravenous injection of MR-CM. Figure 1

## Results

Catheterization of the coronary arteries was successful in all animals under X-ray guidance (n = 13) and MRI guidance (n = 3) and took 15–20 min for X-ray and 30–45 min for MRI fluoroscopy. The perfusion territories of the coronary arteries were defined as hyperenhanced regions on FPP and CE-MRI. The LAD territory was 33.7 ± 2.2% of LV-mass on FPP and 33.0 ± 2.3% on CE-MRI (*P* = 0.63). Bland-Altman analysis showed close agreement between the two methods (0.7 ± 5%). The signal intensity of LAD territory after injection of diluted Gd-based MR contrast media retuned to baseline after 6–8 min on CE MR imaging, suggesting complete washout of the contrast medium from normally perfused myocardium and lack of myocardial damage due to coronary catheterization. DE-MRI demonstrated the infarcted myocardium as hyperenhanced sub-regions of the perfusion territory (7.5 ± 1.2% LV mass) which did not differ from post mortem TTC size (7.1 ± 0.8% LV mass, *P* = 0.99). Postmortem inspection revealed that there was no evidence of vascular or valvular injury caused by the endovascular catheter.

## Conclusion

In this experimental study, we developed a method combining X-ray and MR fluoroscopy for selective mapping of the perfusion territories of the LAD, circumflex and right coronary arteries and quantifying the LAD perfusion territory. The extents of the LAD coronary artery perfusion territory measured on FPP and CE-MRI did not differ and neither did the infarct size on DE-MRI and TTC staining. This experimental method can be used prior to and after local delivery of angiogenic factors or stem cell therapy to determine their efficacy.

